# Supplementary material for: Single or pluralistic? The game and balance of China’s community governance policy tools
Source: PLoS One. 2023 Nov 16;18(11):e0288665. doi: 10.1371/journal.pone.0288665 (PMC10653501; doi:10.1371/journal.pone.0288665)
Supplement: Attachment — Submitted filename: 3.Comments for Manuscript Number PONE-D-23-12250.docx [file pone.0288665.s001.docx]

1. The literature review is incomplete, especially the literature review for the classification of policy tools policy tools. The article only includes competent, learning, persuasive, command, induce,

capacity building, systemic transformation, voluntary, mixed, and coercive. There are other categories that have not been mentioned and it needs to be supplemented.

2. The study selected 163 policy texts including national government work reports, civil affairs development plans, representative provincial government work reports and other policy documents related to community governance. However, the process of screening 163 policies has not been presented. The policy collection process needs to be demonstrated.

3. The text encoding is too simple, please supplement the process of text encoding completely

4. To ensure the reliability of the results, two researchers jointly encode a policy text, the percentage was greater than 70%, it was considered to have high confidence. Is the reliability of this method too low? Maybe need to supplement the explanation of the reliability of this method.

5. The discussion is not closely related to the quantitative analysis of the previous 163 policy texts. The discussion between “The Homogenization of Policy Tools Based on the Criterion of Instrumental Rationality” and “The Trend Towards Diversification of Policy Tools Guided by Value Rationality ”cannot reflect the conclusions drawn from the policy texts well. The analysis content of the policy text is not closely related to the discussion. It is recommended to combine the discussion in the future with the analysis content of the previous policy text.
